# A Lung Cancer Patient Presenting With Gynecomastia: An Uncommon Paraneoplastic Syndrome

**DOI:** 10.7759/cureus.54758

**Published:** 2024-02-23

**Authors:** Ganesh Sanjan, Saikat Banerjee, Ruchi Dua, Prakhar Sharma

**Affiliations:** 1 Department of Pulmonary, Critical Care and Sleep Medicine, All India Institute of Medical Sciences, Rishikesh, Rishikesh, IND

**Keywords:** beta-human chorionic gonadotropin (β-hcg), non small cell lung cancer, gynaecomastia, paraneoplastic, lung cancer

## Abstract

Lung cancer is the most common neoplastic disorder associated with paraneoplastic syndromes. The most common paraneoplastic syndromes are the syndrome of inappropriate release of antidiuretic hormone (SIADH), hypercalcemia of malignancy, ectopic Cushing’s syndrome, and various other neurological syndromes. A few case reports have reported gynecomastia as a paraneoplastic syndrome. Recognition of this uncommon presentation can aid in the early detection of associated malignancies, thus potentially improving outcomes. In this article, we are presenting the case of a male patient in his late sixties who, on presentation, had gynecomastia and was eventually diagnosed with non-small-cell lung cancer (NSCLC).

## Introduction

Paraneoplastic syndromes are a group of clinical disorders that are associated with malignant diseases but are not directly related to mass effects, invasion, or metastasis. They are mostly seen in 10% of lung cancer patients. The most common paraneoplastic disorders include hypercalcemia of malignancy, the syndrome of inappropriate release of antidiuretic hormone (SIADH), ectopic Cushing’s syndrome, and various other neurological, hematological, and rheumatological syndromes. The early recognition of paraneoplastic syndromes may contribute to the detection of a highly treatable, early-stage tumor [1]. Some of these paraneoplastic syndromes, including the one mentioned in this case, may disappear after treatment for the underlying cause.

## Case presentation

Case history and clinical examination

**Figure 1:**
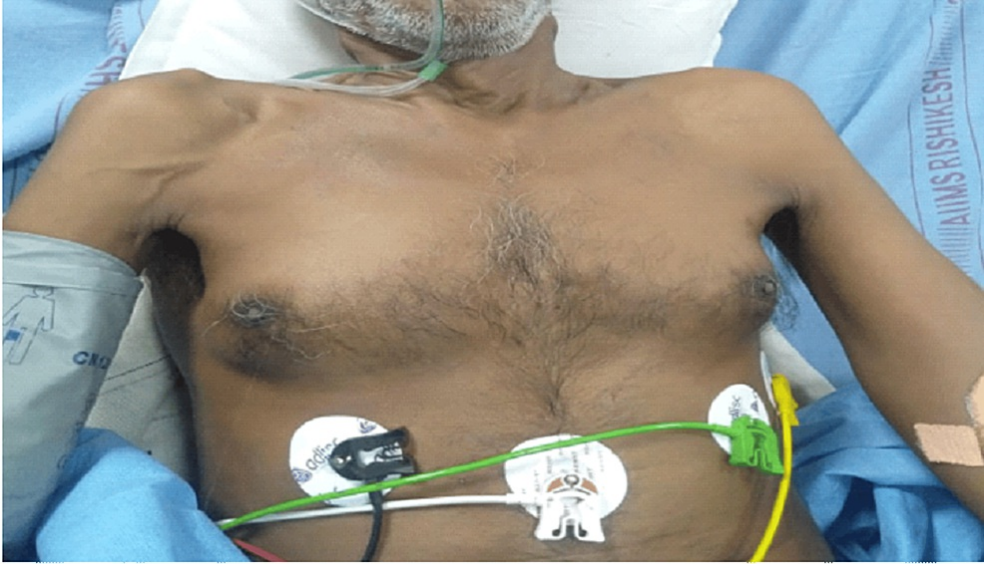
A male smoker in his late sixties presenting with cough, shortness of breath, right chest pain and on examination showing bilateral gynecomastia.

A male patient in his late sixties, with a 40-pack-year smoking history and without any co-morbidities, presented with complaints of dry cough, shortness of breath (SOB), and right-sided chest pain for one month, along with unintentional loss of weight and appetite. The cough was insidious in onset, persistent, and not associated with any diurnal or postural variation. Shortness of breath was also of gradual onset, mMRC (Modified Medical Research Council) grade II, and was persistent. Chest pain was dull, and there were no specific aggravating or relieving factors. There were no accompanying symptoms like hemoptysis, fever, or pedal edema. Vitals were within the normal range. A general examination revealed pallor without any clubbing or lymphadenopathy. On general examination, bilateral gynecomastia was present without any discharge (Figure 1). Except for reduced air entry on the right side, the respiratory examination was normal.

Investigations

**Figure 2:**
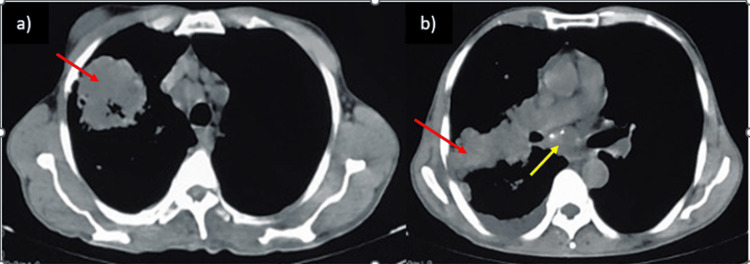
Mediastinal window of high-resolution computed tomography of thorax. (a) Ill-defined necrotic soft tissue mass lesion (red arrow) in apical segment of right upper lobe of 5.9 cm × 5.6 cm × 5.6 cm; (b) medial extension of mass into right hilum and lateral extension to the chest wall (red arrow) is seen along with an enlarged subcarinal mediastinal lymph node (yellow arrow).

The chest X-ray showed a right-sided heterogeneous opacity with lower zone infiltrates. The high-resolution computed tomography (HRCT) thorax revealed an ill-defined necrotic mass lesion in the right upper lobe measuring 5.9 cm × 5.6 cm × 5.6 cm extending into the right hilum (Figure 2a). Mediastinal lymph nodes in the subcarinal location with mild right-sided pleural effusion were also seen (Figure 2b). Screening bronchoscopy revealed no endobronchial growth, and bronchoalveolar lavage was negative for routine infective workup and malignant cytology.

Differential diagnosis

**Figure 3:**
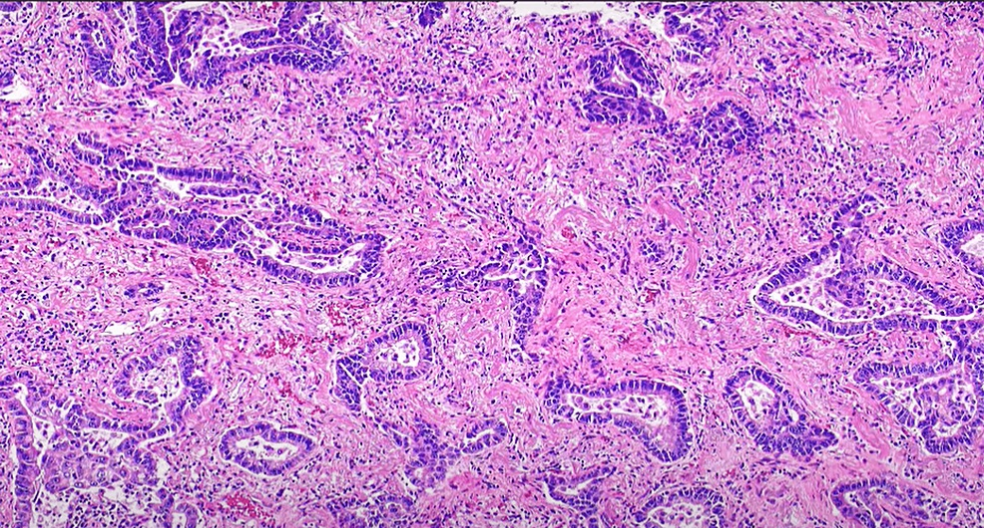
Histopathology of right lung mass section showing acinar cells. Findings suggestive of NSCLC-NOS (p40, TTF-1, Napsin A, Synaptophysin negative in IHC). NSCLC-NOS: non-small-cell lung carcinoma - not otherwise specified, TTF-1: thyroid transcription factor-1, IHC - Immunohistochemistry.

A transthoracic ultrasound-guided biopsy of the right lung mass was done that was suggestive of non-small-cell lung cancer (NSCLC) (Figure 3). Immunohistochemistry (IHC) results were negative for p-40, thyroid transcription factor-1 (TTF-1), Napsin A, and synaptophysin, confirming a diagnosis of NSCLC-NOS (not otherwise specified). Contrast-enhanced computed tomography (CECT) of the abdomen, metastable Technetium 99 labeled methylene diphosphonate (99mTc-MDP) bone scan, and contrast-enhanced magnetic resonance imaging (CEMRI) of the brain done for metastatic workup revealed no evidence of distant metastasis in these regions. However, a diagnostic pleural tap was done, and cytology revealed the presence of atypical cells, suggestive of malignant metastases. The clinical stage of lung cancer was cT3N2M1a (stage IV disease). His performance status was 4 as per the Eastern Cooperative Oncology Group (ECOG). Fine-needle aspiration cytology (FNAC) from the right breast tissue was suggestive of gynecomastia. The urine pregnancy test (UPT) yielded a positive result. The β-subunit of human chorionic gonadotropin (β-hCG) was elevated at 639 mIU/ml (normal value <5 mIU/ml). His testicular ultrasound was normal. There was no relevant drug history and no history of hypogonadism. Furthermore, in his liver and renal function tests, serum levels of prolactin, testosterone, luteinizing hormone, follicle-stimulating hormone, thyroid-stimulating hormone, and cortisol were within the normal range. So, we arrived at the diagnosis of paraneoplastic syndrome associated with lung cancer.

Treatment

After 72 hours of admission to our ward, his clinical condition deteriorated with a new-onset fever and a flare-up of cough, SOB, and chest pain. Repeat chest X-rays showed new infiltrates, suggesting hospital-acquired pneumonia. He was shifted to an intensive care unit in view of acute hypoxemic respiratory failure. He was managed first on non-invasive ventilator support and then intubated due to worsening sensorium.

Outcome and follow-up

He was deemed unfit for any definitive therapy for malignancy because of the ongoing respiratory infection and poor performance status. He succumbed to his illness on day 7 of hospitalization as a result of hypoxemic respiratory failure and septicemia due to hospital-acquired pneumonia.

## Discussion

Lung cancer patients can present with paraneoplastic syndrome as their first clinical manifestation. The various mechanisms of paraneoplastic syndromes include the ectopic production of hormones and cytokines and the excessive release of products released by the tumor. Gynecomastia in lung cancer is due to excessive secretion of β-hCG. The histology of these tumors is usually adenocarcinoma. A few cases of squamous cell carcinoma and small-cell lung carcinoma have also been reported. Gynecomastia affects 3% of cancer patients at the time of diagnosis. Gynecomastia as a paraneoplastic syndrome has been reported in about 2.4% of lung cancer patients [2].

The evaluation of gynecomastia should start by first ruling out its common causes. A detailed drug history, including androgen therapy in hypogonadal men and chronic liver or renal disease, should be ruled out. β-hCG levels should be checked, and if above normal, the search should include β-hCG-secreting trophoblastic and testicular tumors. Non-trophoblastic tumors of the gastric, liver, and kidney can also cause gynecomastia with ectopic production of β-hCG. Out of these, lung cancers are the most common cause of hCG secretion. Complete resolution of gynecomastia after resection/treatment of the lung cancer, along with changes in the level of the hCG β subunit, confirm the relationship between gynecomastia, lung tumor, and β-hCG levels. In our case, hormonal evaluation, liver, and kidney function tests were all in the normal range. UPT was positive, and serum β-hCG levels were high. Histopathology was suggestive of NSCLC as the potential etiology.

We conducted a literature search in PubMed, Embase, and Cochrane databases with a Boolean approach using the terms “lung cancer," “paraneoplastic syndrome,” and "gynecomastia." We found 17 case reports and two case series. However, there were no cohort or case-control studies. All case reports in the English language whose full abstract/full text were available were included. Abstracts of eight studies were not found as they were old, and one study was excluded as it was in Spanish and the English version could not be obtained. This literature review provides an overview of gynecomastia in patients with lung cancer. Most of the studies showed that gynecomastia resolved after starting treatment for primary lung malignancies.

Baez et al. [3] reported a case of a 43-year-old male with a one-month history of shortness of breath, dry cough, hemoptysis, and weight loss. A physical examination was relevant for the presence of gynecomastia without discharge. A computed tomography (CT) scan of the thorax, abdomen, and pelvis revealed a 7-cm left upper lobe lung and enlarged mediastinal lymph nodes. A bronchoscopy-guided biopsy of the left upper lobe mass was suggestive of poorly differentiated squamous cell carcinoma. Serum β-hCG levels were found to be elevated to 326.2 mIU/ml (normal ≤ 0.5 mIU/ml). The patient’s course was complicated by respiratory failure secondary to aspiration and evidence of metastatic brain disease.

Lazopoulos et al. [2] reported gynecomastia and galactorrhea as paraneoplastic syndromes in a 62-year-old male. A chest X-ray revealed a left upper lobe tumor. The patient had high levels of serum β-hCG and prolactin. The pathology report showed poorly differentiated adenocarcinoma (T2N1M0). The patient received chemotherapy with cisplatin and vinorelbine. After one year, there was a regression of both gynecomastia and mastodynia. Serum β-hCG levels normalized, and there was no nipple discharge.

Goyal et al. [4] reported the case of a 60-year-old man with a productive cough and dyspnea of three months duration. There was bilateral gynecomastia with watery discharge from the nipples. A CT of the chest showed a mass lesion measuring 9.9 cm × 9.4 cm in the right upper lung lobe, with enlarged mediastinal lymph nodes. An endobronchial biopsy showed squamous cell carcinoma. Immunohistochemistry for β-hCG was positive and negative for estrogen and testosterone. Serum β-hCG was 20,000 IU/L. Chemotherapy (paclitaxel 175 mg/m² and cisplatin 65 mg/m² each on day 1 of the three-weekly cycle) was started, following which gynecomastia decreased in size and galactorrhea also disappeared. Table 1 lists some other relevant case reports.

**Table 1:** List of other case reports of lung cancer with gynecomastia. β-hCG: beta subunit of human chorionic gonadotropin.

Author	Year of publication	Age/sex	Histology	Serum β-hCG (mIU/ml)	Origin
Cirit Koçer et al. [5]	2016	43/Male	Non-small-cell lung cancer	4261	Turkey
Okutur et al. [6]	2010	50/Male	Pleomorphic carcinoma	6500	Turkey
Ahmed et al. [7]	2005	43/Male	Large-cell carcinoma	2125	USA
Yaturu et al. [8]	2002	51/Male	Large-cell carcinoma	98	Louisiana
Bustamante et al. [9]	1995	30/Male	Giant-cell carcinoma	-	Germany
Metz et al. [10]	1978	51/Male	Large-cell carcinoma	109	Washington
Fairlamb and Boesen [11]	1975	67/Male	Small-cell lung cancer	-	London

After reviewing all articles, we found that most of the case reports were with NSCLC, particularly adenocarcinoma, and had bilateral gynecomastia, as seen in our case. However, in rare cases, unilateral gynecomastia has also been mentioned.

## Conclusions

This case highlights the importance of including β-hCG as a part of the routine workup of paraneoplastic syndromes in lung cancer in addition to investigations like serum calcium and sodium. The patient presented to us with gynecomastia, and β-hCG levels turned out to be high. In such cases, the presence of non-trophoblastic tumors should also be considered in addition to testicular tumors and other trophoblastic tumors. This is reflected in this case, in which, as a rare occurrence, the diagnosis was non-small cell lung cancer.

Hence, it is important that when an elderly male patient presents with gynecomastia and/or galactorrhea with a relevant clinical history, one should consider the possibility of lung cancer after ruling out other common causes. Actively looking for cancer may help in the early detection of disease and improve disease outcomes. In our patient, the patient already had metastatic stage disease when he presented to us and unfortunately succumbed to his illness after a few days of hospitalization.

## Appendices

Patient relative's perspective

Watching my father deal with the continuous cough, breathlessness, and chest pain was hard. All of us could see the worry in his eyes and feel the struggle every day. We felt helpless, wanting to fix it but not knowing how. We were hoping it was nothing serious and prayed for the doctors to make it better. But as time passed, our worry just grew. Each day felt heavier, filled with uncertainty. The doctor informed me that my father was most likely suffering from an advanced stage of lung cancer and that the road ahead was not going to be very smooth. Yet, we held onto this tiny hope that things would turn around and that my father would get better. The doctors tried hard, and he was eventually put on ventilatory support; however, in just a week, everything changed. The end came so suddenly, leaving us all in shock. Losing my father hurts so much. We wish we had more time and wish things had turned out differently. There is this emptiness now, a void that is hard to fill. We will always remember the struggle, the hope we held onto, and the sudden loss. Life can be really tough sometimes, and this was one of those times.
